# The Structure and Nucleotide-Binding Characteristics of Regulated Cystathionine β-Synthase Domain-Containing Pyrophosphatase without One Catalytic Domain

**DOI:** 10.3390/ijms242417160

**Published:** 2023-12-05

**Authors:** Ilya M. Zamakhov, Viktor A. Anashkin, Andrey V. Moiseenko, Victor N. Orlov, Natalia N. Vorobyeva, Olga S. Sokolova, Alexander A. Baykov

**Affiliations:** 1 Belozersky Institute of Physico-Chemical Biology, Lomonosov Moscow State University, 119899 Moscow, Russiavictor_anashkin@belozersky.msu.ru (V.A.A.);; 2 Department of Chemistry, Lomonosov Moscow State University, 119899 Moscow, Russia; 3 Department of Biology, Lomonosov Moscow State University, 119899 Moscow, Russiasokolova@mail.bio.msu.ru (O.S.S.); 4 Department of Biology, Shenzhen MSU-BIT University, Shenzhen 518172, China

**Keywords:** electron microscopy, diadenosine tetraphosphate, Förster resonance energy transfer (FRET), (methyl-anthraniloyl)-adenosine-5´-monophosphate (Mant-AMP), ThermoFluor, cryo-electron microscopy, thermal unfolding, domain swapping, cystathionine β-synthase domain

## Abstract

Regulatory adenine nucleotide-binding cystathionine β-synthase (CBS) domains are widespread in proteins; however, information on the mechanism of their modulating effects on protein function is scarce. The difficulty in obtaining structural data for such proteins is ascribed to their unusual flexibility and propensity to form higher-order oligomeric structures. In this study, we deleted the most movable domain from the catalytic part of a CBS domain-containing bacterial inorganic pyrophosphatase (CBS-PPase) and characterized the deletion variant both structurally and functionally. The truncated CBS-PPase was inactive but retained the homotetrameric structure of the full-size enzyme and its ability to bind a fluorescent AMP analog (inhibitor) and diadenosine tetraphosphate (activator) with the same or greater affinity. The deletion stabilized the protein structure against thermal unfolding, suggesting that the deleted domain destabilizes the structure in the full-size protein. A “linear” 3D structure with an unusual type of domain swapping predicted for the truncated CBS-PPase by Alphafold2 was confirmed by single-particle electron microscopy. The results suggest a dual role for the CBS domains in CBS-PPase regulation: they allow for enzyme tetramerization, which impedes the motion of one catalytic domain, and bind adenine nucleotides to mitigate or aggravate this effect.

## 1. Introduction

Numerous biosynthetic reactions critically depend on the ability of cells to degrade pyrophosphate formed from ATP as a byproduct [1]. In most cell types, this function is performed by specific soluble enzymes, pyrophosphatases (PPases; E.C. 3.6.1.1), belonging to three nonhomologous families [2,3,4]. Family II PPases, found in prokaryotes and archaea, surpass other PPases by catalytic efficiency (*k*_cat_ of up to 10^4^ s^−1^), and their prevailing form is a dimer of identical subunits, each formed by two catalytic domains, DHH and DHHA2 [3]. The active site is located between the domains in each subunit and binds one transition metal ion (Mn^2+^ or Co^2+^) and 1–2 Mg^2+^ ions as essential activators and the MgPP_i_ complex as a substrate.

**Figure 1:**
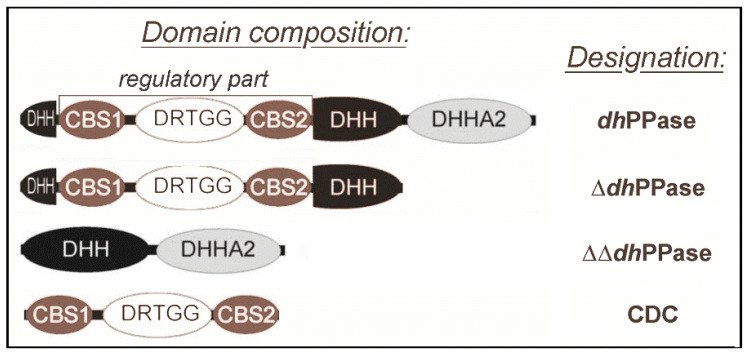
Domain organization of the full-size CBS-PPase of *Desulfitobacterium hafniense* (*dh*PPase) and its deletion variants used in this study. DHH and DHHA2 are catalytic domains, and the CBS1, CBS2, and DRTGG domains comprise a regulatory insert within the DHH domain.

In some organisms, the two catalytic domains of family II PPases are supplemented by a pair of cystathionine β-synthase (CBS) domains and one DRTGG domain that together form a regulatory insert within the DHH domain [3,5] (Figure 1). Such CBS-PPases are less efficient catalysts (*k*_cat_ is less by 1–2 orders of magnitude) because the regulatory insert acts as an internal inhibitor. AMP and ADP binding to the CBS domains further increases the inhibition [5], whereas ATP and diadenosine polyphosphate binding alleviates it [5,6], allowing efficient regulation of enzyme activity and, hence, cellular PP_i_ level and the intensity of biosynthesis by the energy status of the cell. Quite important in this respect is the unprecedented affinity of CBS-PPase for diadenosine tetraphosphate (Ap_4_A), a cellular stress alarmone [7]. Its accumulation under stress conditions and concomitant CBS-PPase activation provide the host organism with a way to escape stress by decreasing cellular PP_i_ levels to restore biosynthesis [6]. No other protein with or without the CBS domain demonstrates similar affinity for Ap_4_A, supporting the key role of CBS-PPase in combatting stress in host bacteria.

CBS domains are relatively small (approximately 60 amino acid residues) units that are usually present in pairs with a characteristic interaction between them [8]. They are found in thousands of soluble and membrane-associated proteins of all kingdoms of life [9] and have a regulatory function in all characterized cases [8,10]. The regulatory ligands are commonly, but not exclusively, various adenosine phosphates. Mutations in CBS domains are associated with several hereditary diseases in humans [11]. CBS domains can be excised from proteins in a functionally competent form; furthermore, some proteins with similar structure and function either have or do not have CBS domains, depending on the host organism [12,13,14,15]. Crystallization of CBS domain-containing proteins is difficult, likely because of their structural flexibility, and most available structures are for CBS domain-only proteins or fragments of multidomain proteins. This explains why only limited information is available on the mechanism of CBS domain-mediated regulation.

In this context, CBS-PPase provides a useful model for studying this mechanism. CBS-PPase regulation has been thoroughly characterized using kinetic and site-directed mutagenesis approaches. The regulation depends on multiple effects, including changes in *k*_cat_ and substrate binding cooperativity [15]; regulatory ligand binding is also cooperative [15]. Therefore, the regulatory phenomenon should depend on a branched network of interactions that link the regulatory and active sites within a subunit and with other subunits in the tetrameric protein molecule.

Further advances in understanding CBS-PPase regulation critically depend on the knowledge of its 3D structure. Previous X-ray crystallographic studies have elucidated the structures of the DHH and DHHA2 domains in dimeric CBS domain-lacking “canonical” Family II PPase [16,17] and the structure of a dimeric separate regulatory part of CBS-PPase [18]. The first proposed model of the entire CBS-PPase molecule based on these structures also assumed that it is homodimeric [18]. However, more recent studies have demonstrated that CBS-PPase is tetrameric [19,20]. We have subsequently proposed two possible structural models of the domain organization in tetramers [19], but neither of them has been verified experimentally.

In the present study, we combined in silico and in vitro approaches to determine the 3D structure of CBS-PPase without the DHHA2 domain, the expected most movable part of the protein molecule. The inactive truncated enzyme variant (Δ*dh*PPase) retained the oligomeric structure of the full-size enzyme and its ability to bind adenosine phosphates, highlighting the role of the deleted domain in the regulatory mechanism.

## 2. Results

The DHHA2 domain is the most movable element in unregulated Family II PPases [16,17]. Therefore, we deleted this domain from CBS-PPase in order to obtain a more stable structure. Because the deleted DHHA2 domain contains an important part of the active site, the Δ*dh*PPase variant did not catalyze PP_i_ hydrolysis. Therefore, to evaluate the functional competence of Δ*dh*PPase and, hence, its structural integrity, we measured its interaction with regulatory ligands. We also determined the oligomeric structure of Δ*dh*PPase and analyzed its thermal unfolding in the absence and presence of the ligands. Finally, we determined the shape of the deletion variant and computationally predicted its detailed 3D structure.

### 2.1. Nucleoside Phosphate (Ap_4_A and AMP) Binding

**Figure 2:**
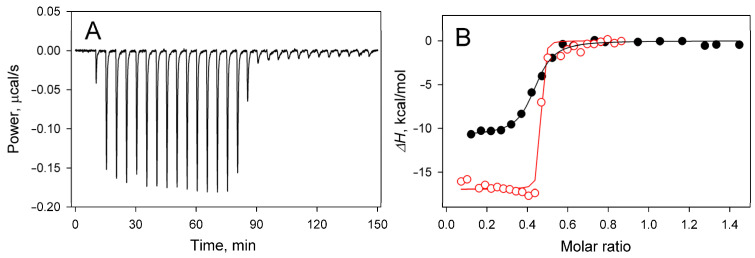
ITC measurements of Ap_4_A binding to full-size *dh*PPase and its deletion variant. (**A**) Typical raw data for successive injections of Ap_4_A into Δ*dh*PPase solution. (**B**) Integrated heats for titration of Δ*dh*PPase (open circles) or full-size *dh*PPase (closed circles) by Ap_4_A after correction for dilution. The titration curve for the full-size *dh*PPase is very similar to that reported previously [6]. The lines show the best fits to a “single-binding-site” model.

Binding measurements were attempted with two regulating adenosine phosphates—diadenosine tetraphosphate (Ap_4_A, activator) and AMP (inhibitor)—using isothermal titration calorimetry (ITC). Figure 2 presents the ITC data for Ap_4_A.The titration curve obtained for the full-size *dh*PPase (Figure 2B, black symbols) was quite similar to that reported earlier [6] and demonstrated tight binding with a stoichiometry of approximately one Ap_4_A molecule per two subunits (Table 1) or two Ap_4_A molecules per tetramer. Consistent with this stoichiometry, the two adenosine groups of the Ap_4_A molecule form contacts with both subunits in the structure of a separate dimeric regulatory part (CDC in Figure 1) of *C. perfringens* CBS-PPase [18]. Notably, the stoichiometry was twice as large, and the enthalpy change Δ*H* was two times smaller in a similar titration of *dh*PPase with AMP (Table 1). The variant Δ*dh*PPase exhibited the same Ap_4_A-binding stoichiometry but a much sharper transition (i.e., stronger binding affinity) and a larger enthalpy change Δ*H* (Table 1). However, the binding constants could only be roughly estimated from Figure 2B because of the very tight binding (Table 1). Nevertheless, the Δ*dh*PPase affinity was undoubtedly significantly higher, and the difference was reproducible in replicate titrations.

Similar titrations attempted with AMP were unsuccessful because of the incipient precipitation of AMP-bound Δ*dh*PPase. Precipitation (loss of solution transparency) started after two or three AMP additions, resulting in an unusual titration curve with an endothermic and exothermic signals upon each addition. Notably, precipitation was not observed with full-size *dh*PPase, which is consistent with earlier data [6]. The heat effects of the first two AMP additions, which did not cause Δ*dh*PPase precipitation, were two times less and similar to those previously reported for full-size *dh*PPase [6]. This finding and the precipitation effect indicated that Δ*dh*PPase can bind AMP.

**Figure 3:**
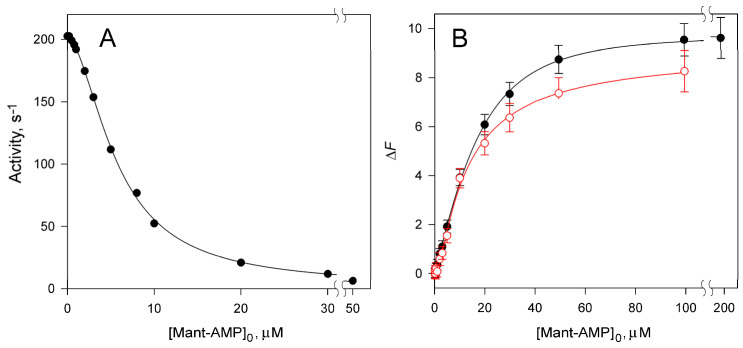
Mant-AMP binding to full-size *dh*PPase and its deletion variant. (**A**) Mant-AMP inhibition of *dh*PPase activity. The line shows the best fit of Eqns. 2–6 and was obtained using the parameter values found in Table 2. (**B**) FRET titration of Δ*dh*PPase (open circles, red line) and *dh*PPase (closed circles, black line) with Mant-AMP. The lines show the best fits of Equations (1)–(5) and were obtained using the parameter values in Table 3.

**Scheme 1:**
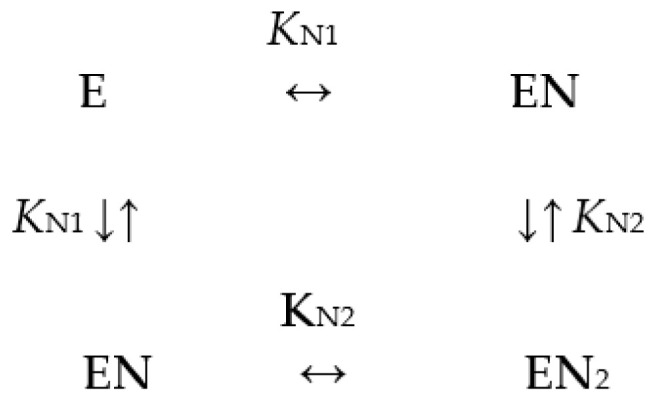
Nucleotide binding to two sets of regulatory sites of CBS-PPase.

Quantitative characteristics of inhibitor binding were instead obtained for Mant-AMP, an AMP derivative containing a fluorescent N-methyl-anthraniloyl substituent in the ribose moiety. Mant-AMP binding to the full-size *dh*PPase was first characterized by measuring its effect on enzymatic activity (Figure 3A). The inhibition curve was clearly sigmoidal and was, therefore, analyzed in terms of Scheme 1, which assumes the presence of two types of interacting binding sites for Mant-AMP on the enzyme. A similar analysis of *dh*PPase inhibition by AMP has been performed previously under identical conditions [15], and Table 2 compiles the parameter values for both nucleotides. Their comparison indicates that both nucleotides inhibited *dh*PPase almost completely and exhibited strongly positive binding cooperativity (the Hill coefficient of approximately 1.7, the *K*_i1_/*K*_i2_ ratio of approximately 50). The major difference was a fourfold greater *K*_i_ value for Mant-AMP, indicating its lower binding affinity. These results demonstrated a similar behavior of the two nucleotides as *dh*PPase ligands.

Mant-AMP binding to the inactive Δ*dh*PPase variant was measured fluorimetrically in the absence of the substrate, and parallel data were obtained for the full-size *dh*PPase. To suppress background fluorescence, bound Mant-AMP was excited by energy transfer (FRET) from a nearby Trp residue that is excited at a much lower wavelength. FRET titration could be performed at a lower protein concentration and faster than ITC titration (1.5 versus 5 min per point) to diminish precipitation. To further decrease the overall time of Δ*dh*PPase contact with Mant-AMP, a titration curve was constructed from two overlapping titrations performed separately with identical protein samples but using different Mant-AMP concentration ranges (0.2–10 and 10–100 µM). Together with the lower propensity of Mant-AMP to induce protein aggregation, these precautions eliminated the protein precipitation problem with Δ*dh*PPase.

The titration curves for *dh*PPase and Δ*dh*PPase (Figure 3B) look similar, yielding very similar parameter values (Table 3). Mant-AMP binding to both *dh*PPase forms demonstrated significant positive cooperativity (*K*_N1_ > *K*_N2_). Of note, AMP binding to *dh*PPase is also a positively cooperative process [15]. Considering that both the *K*_i_ values in Table 2 and *K*_N_ values in Table 3 are true binding constants, the differences between them for *dh*PPase suggested that substrate bound to active sites in *K*_i_ measurements significantly altered nucleotide binding to regulatory sites. Specifically, the bound substrate increased the positive binding cooperativity (*K*_i1_/*K*_i2_ > *K*_N1_/*K*_N2_), mainly via the binding of the second ligand molecule (*K*_i2_).

### 2.2. Effects of Deletion and Nucleotide Binding on Protein Thermal Stability

**Figure 4:**
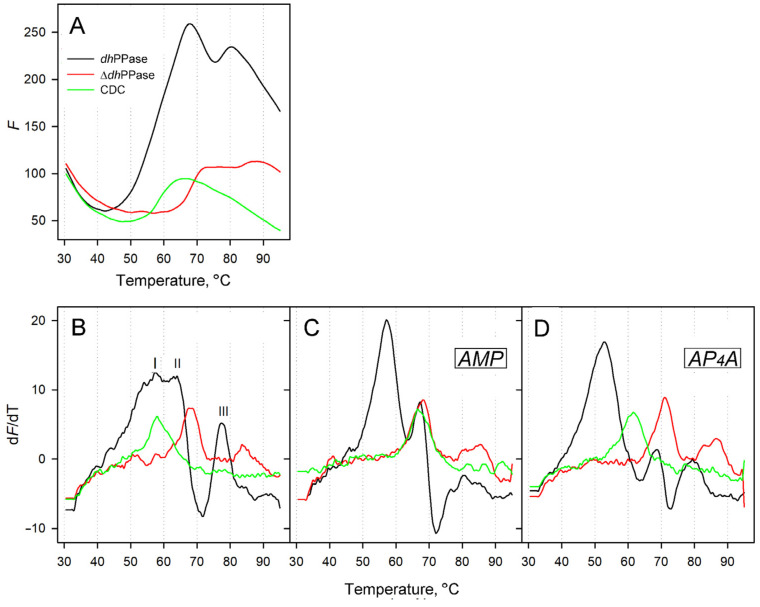
Effects of adenosine phosphates on the thermal stability of full-size *dh*PPase and its two deletion variants as monitored by SYPRO Orange fluorescence. (**A**) Representative original melting profiles for full-size *dh*PPase and its deletion variants in the absence of adenosine phosphates. (**B**–**D**) First derivatives of the fluorescence versus temperature dependencies measured in the absence of adenosine phosphates (**B**), in the presence of AMP (**C**), and in the presence of Ap_4_A (**D**). Color codes for the enzyme variants are indicated in panel A. The major peaks for the full-size enzyme are marked as I, II, and III in panel (**B**). The curves in panels (**B**–**D**) are the averages of three independent runs.

The effects of domain deletion and nucleotide binding on the stability of the protein molecule were assessed by measuring its thermal unfolding profile. In the method often called ThermoFluor [21], protein unfolding under the action of an applied temperature gradient is monitored with a dye whose fluorescence increases upon binding to the hydrophobic groups that become exposed in the unfolded or partially unfolded protein (Figure 4A). The principal characteristic derived from a ThermoFluor profile is the melting temperature (*T*_m_), which is determined as the peak temperature on the first derivative plot d*F*/dT (Figure 4B–D). In a multidomain protein, such as CBS-PPase, separate structural units unfold sequentially, resulting in several peaks on the d*F*/dT plot. For reliable peak assignments to specific domains, two other available deletion variants of *dh*PPase, comprising the separate catalytic or regulatory part, were used (ΔΔ*dh*PPase and CDC in Table 1). Importantly, no protein precipitation was observed in the ThermoFluor measurements because of the 10-fold shorter duration of the experiment and lower protein concentration compared with the ITC titration.

The first-derivative plot for the full-size *dh*PPase demonstrated three major peaks with *T*_m_ values of 57.0, 64.0, and 77.5 °C (Figure 4B), likely corresponding to three structural units—the DHHA2 domain, the regulatory part (two CBS and one DRTGG domains), and DHH domain, respectively. All these units preserve their structures when separated from the protein [15,17,18] and are, therefore, self-contained and should unfold independently. The deletion of the DHHA2 domain eliminated peak I (Figure 4B), suggesting that it corresponds to this domain. This assignment is consistent with the presence of peak I in the melting profile of the separate catalytic part (ΔΔ*dh*PPase) (Table 4).

Peak II likely belongs to the regulatory part, as it is found in the melting profiles of both Δ*dh*PPase and CDC but not ΔΔ*dh*PPase (Table 4). This assignment is also supported by the observations that AMP and Ap_4_A universally increased *T*_m_ for separate CDC and for peak II of *dh*PPase and Δ*dh*PPase (Table 4). In contrast, ligand effects on peak I in *dh*PPase were different—AMP had little effect on *T*_m_, whereas Ap_4_A decreased it appreciably (Table 4). The different effects of AMP (inhibitor) and Ap_4_A (activator) on DHHA2 stability correlated with their opposite effects on enzymatic activity, and this observation may provide a clue to the mechanism of activity regulation. Peak III is absent only in CDC and likely belongs to the DHH domain. Consistent with this assignment, the melting profile of homologous Family II PPase from *Streptococcus gordonii*, formed by only the DHHA2 and DHH domains [22], exhibited only two peaks with *T*_m_ values of 56.5 and 82.3 °C. These values clearly match peaks I and III of *dh*PPase and the two peaks of ΔΔ*dh*PPase.

### 2.3. Oligomeric Structure of ΔdhPPase

**Figure 5:**
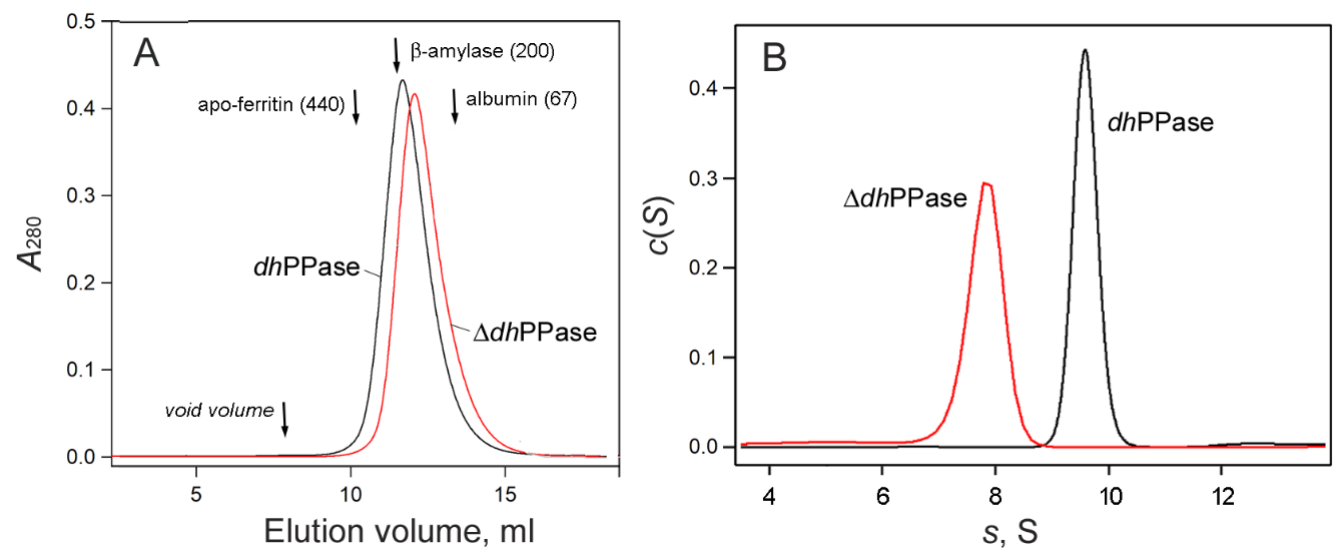
Profiles of the size exclusion chromatography (**A**) and analytical sedimentation (**B**) of the full-size *dh*PPase and its deletion variant lacking the DHHA2 domain. The void volume and elution volumes of the three marker proteins (with masses in kDa shown in parentheses) are indicated by arrows in panel A.

Wild-type CBS-PPases, including *dh*PPase, are homotetrameric [19,20]. The deletion variant, which lacked the catalytic DHHA2 domain, retained the tetrameric structure, as demonstrated by two different approaches—size-exclusion chromatography and analytical ultracentrifugation. The elution volumes of native *dh*PPase and Δ*dh*PPase in size-exclusion chromatography differed by 0.35 mL (Figure 5A), corresponding to a mass difference of ~38 kDa. This value matches reasonably well the molecular mass of the deleted domain (13.9 kDa) multiplied by four. The deletion decreased the sedimentation coefficient, *s*_20,w_, of *dh*PPase by only 0.8 S, again consistent with the tetrameric structure of its deletion variant. The molecular masses estimated from these measurements are summarized in Table 5.

### 2.4. Single-Particle Electron Microscopy of ΔdhPPase

Cryo-electron microscopy (cryo-EM) was used to determine the domain organization in Δ*dh*PPase. Although a recent attempt to use this approach with full-size *dh*PPase was unsuccessful [24], the deletion variant, lacking the movable DHHA2 domain, demonstrated more promising results.

Before cryo-EM grid preparation, sample monodispersity and aggregation were assessed using negative-stain transmission electron microscopy. The resulting micrographs showed good particle distribution (Figure S1), and the resulting reference-free class averages clearly revealed a symmetrical linear structure, despite some heterogeneity. The small deviations from linearity seen in some images may be an artifact of the molecule binding to the solid support.

**Figure 6:**
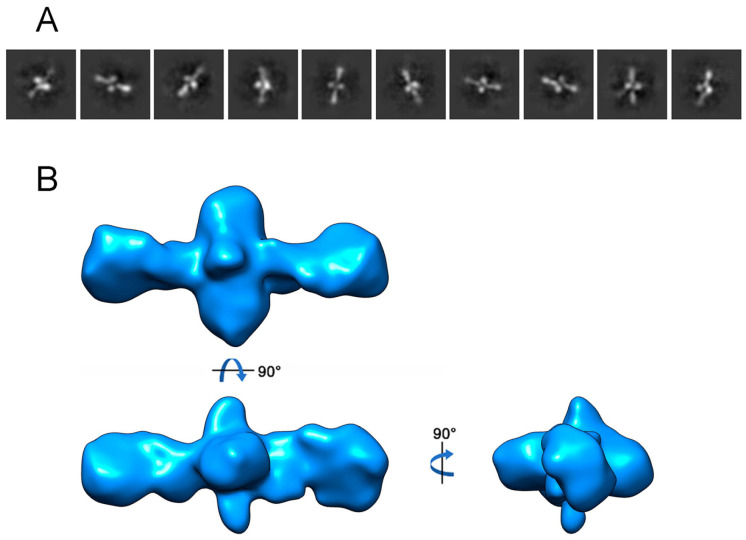
The 3D structure of tetrameric Δ*dh*PPase determined by cryo-EM. (**A**) Representative 2D class average images of Δ*dh*PPase. (**B**) Three views of the 16 Å Δ*dh*PPase single particle reconstruction generated from the cryo-EM data.

To assess the spatial organization of the Δ*dh*PPase tetramer, cryo-EM was applied to the same enzyme sample. The 2D classification yielded approximately 15,000 single particle projections that produced good quality class averages (Figure 6A). The angular distribution of particle projections reveals that the majority of projections depict views perpendicular to the longest dimension of the protein (Figure S2). The 3D reconstruction and non-uniform refinement led to the density map with 16 Å resolution (Figure 6B).

### 2.5. The Modeled 3D Structure of ΔdhPPase

**Figure 7:**
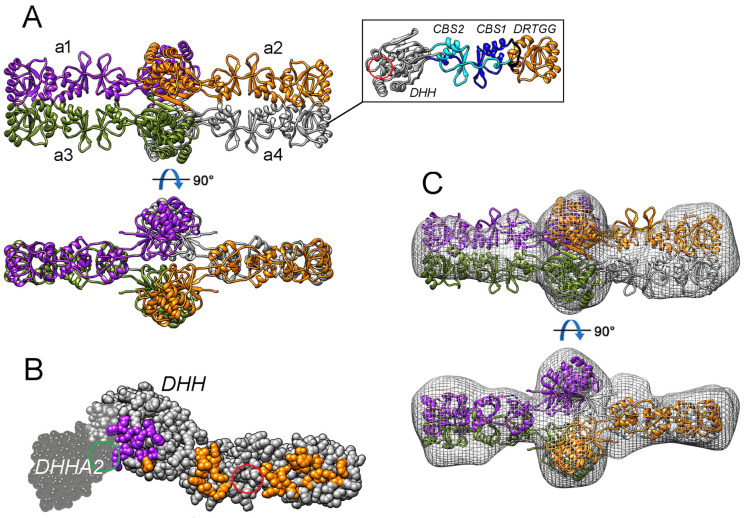
The 3D structure of tetrameric Δ*dh*PPase predicted by Alphafold2. (**A**) Two views of the modeled structure in a ribbon representation with differently colored subunits marked as a1, a2, a3, and a4 in one view. The side panel shows a separate subunit a4 with differently colored domains. The region of the half of the active site retained in the truncated subunit is indicated by a red circle. (**B**) Subunit contact regions. The panel shows subunit a4 with missing DHHA2 domain in an open conformation; the DHHA2 domain was excised from the structure of a homologous PPase of *Bacillus subtilis* [16] and manually added to the Δ*dh*PPase subunit. The colored surface residues form contacts with subunit a1 (violet) and subunit a2 (orange). The green and red circles mark the regions of the active and regulatory sites, respectively. (**C**) Two views of the modeled structure of tetrameric Δ*dh*PPase accommodated within the cryo-EM-generated density map. The figure was created with UCSF Chimera [26].

We previously used Modeller to propose two possible models of tetrameric full-size apo-*dh*PPase [19]. Here, we predicted the Δ*dh*PPase apo-structure using an advanced tool, Alphafold2 [25]. The principal feature of the modeled Δ*dh*PPase structure is that four DHH domains form a core, whereas the regulatory parts are positioned in the outmost region from the core region (Figure 7A). The modeled structure displays D2 symmetry (Figure S3).

According to the modeled structure, each subunit contacts two other subunits in tetrameric Δ*dh*PPase, forming four contacts with one of them (through DRTGG, CBS1, CBS2, and DHH domains, marked orange) and one contact with the other (through DHH domain, marked violet) (Figure 6B). The DHH domains of subunits a1 and a4 and, similarly, subunits a2 and a3 interact by forming common β-sheets with a 790-Å^2^ buried surface area per subunit in each pair. This type of DHH domain interaction with a similar buried surface area of 805 Å^2^ per subunit was previously observed in the crystal structure of canonical dimeric Family II PPases, which lack regulatory domains [16,17]. Each DHH domain also forms an alternative interaction in the pairs a2–a4 and a1–a3, but these alternative contacts appear to be much weaker, based on the buried surface areas of only 112 Å^2^ per subunit.

However, the principal tetramer-stabilizing interaction occurs via the regulatory domains within the pairs a1–a3 and a2–a4, as in the crystal structure of the dimerized regulatory part of *Clostridium perfringens* CBS-PPase [18]. The buried surface area for the entire regulatory part comprising CBS1, CBS2, and DRTGG domains in Δ*dh*PPase is 2110 Å^2^ per subunit, by far surpassing all other contacts. In the dimerized separate regulatory part of *C. perfringens* CBS-PPase, this contact area was of similar size (1770–2190 Å^2^ per subunit) [18]. The modeled structure was accommodated with a correlation factor of 0.86 within the density map obtained by cryo-EM (Figure 7C).

## 3. Discussion

### 3.1. 3D Structure of dhPPase

The unusual flexibility of the CBS-PPase structure apparently explains the failure of previous attempts by several groups to crystallize the full-size enzyme for X-ray structural analysis. A recent breakthrough in the 3D structure prediction of oligomeric proteins [25] allowed us to construct a reliable model of its less flexible deletion variant. The validity of the predicted structure is supported by the fact that it is grounded on the known crystal structures of its two parts derived from highly homologous proteins—the two-domain “canonical” Family II PPase and the regulatory part of *C. perfringens* CBS-PPase. Moreover, the predicted nontrivial shape of the Δ*dh*PPase molecule was directly confirmed by single-particle electron microscopy. Both types of evidence refer to the deletion variant of CBS-PPase, which lack one catalytic domain, but is likely also applicable to the full-size enzyme. This premise is supported by data showing that the structures of both enzyme forms are tetrameric and fully functional in their regulatory parts (capable of high-affinity AMP and Ap_4_A binding). The tentative model of the full-size *dh*PPase deduced from small-angle X-ray scattering data [24] is entirely different from that in Figure 6, perhaps, because of the limitations of this method.

The major subunit contacts stabilizing the Δ*dh*PPase tetramer are those between the regulatory parts (two such interactions per tetramer) and between the common β-sheet-forming DHH domains (two more interactions per tetramer). These interactions are intertwined to form tetramers, although they form only dimers separately [16,17,18]. The Δ*dh*PPase structure presents an unusual type of domain swapping [27], wherein the domains are partially exchanged not between monomers, as in the classical case, but between dimers. To the best of our knowledge, identical architecture has not been observed in other tetrameric CBS-proteins. An “inverted” type of linear homotetramer was found in *Mycobacterium tuberculosis* cystathionine β-synthase [28] (7XNZ) and the protein THA0829 from *Thermus thermophilus* HB8 [29] (5AWE) with four CBS modules in the central part and two peripheral catalytic domain dimers on both sides. The basal form of IMP dehydrogenase is a planar tetramer in which only catalytic domains interact with one another [30] (1ZFJ), but ATP and GTP induce the combination of two planar tetramers into an octamer along the fourfold axis via intertwined interactions of both catalytic and CBS domains [31,32,33] (3DQW, 7OJ1, and 7PJI, respectively). GMP reductase forms a similar octamer [34].

### 3.2. Mechanism of Regulation

The differential effects of adenosine phosphates on the activities of CBS domain-containing enzymes are usually interpreted in terms of internal inhibition caused by the CBS domains [11,12,15,35]. According to this concept, evolutionarily acquired CBS domains distort the protein structure, thereby suppressing its functionality, and CBS-domain ligands either mitigate or aggravate these effects by appropriately tuning the protein conformation. In both cases, the involved conformational change consumes part of the ligand-binding energy and creates structural tension. Because the catalytic and regulatory sites are separated in space, signal (tension) transfer between them should involve the intermediate element(s) of the protein structure. Accordingly, deletion of the involved structural element(s) should ease the tension and stimulate ligand binding. In the absence of regulatory ligands, substrate binding induces a catalytically competent active site conformation, causing structural tension that consumes part of the binding energy. This logic may explain, with some reservations, why AMP increases and Ap_4_A decreases the accessibility of the *dh*PPase active site for substrate, as characterized by the Michaelis constant [36], and why this parameter is greater for *dh*PPase in the absence of adenosine phosphates than for its CBS domain-lacking ΔΔ*dh*PPase variant [15].

The tetrameric structure appears to be another important feature of CBS-PPase, allowing for its regulation by adenosine phosphates. The catalytic DHH and DHHA2 domains are connected by a flexible linker in CBS-PPase, permitting closure and opening of the active site, located in between, during the catalytic cycle. In dimeric canonical Family II PPase, which lacks the regulatory part, subunits interact via their DHH domains, whereas DHHA2 domains have a large freedom to move [16,17]. In tetrameric CBS-PPase, whose catalytic core is formed by four DHH and four DHHA2 domains, the motion of the latter domain is more constrained, resulting in internal inhibition. We speculate that the activity of CBS-PPase is regulated by changes in DHHA2 domain mobility, and the effect of DHHA2 deletion on Ap_4_A binding provides support for this assertion. The increased affinity of Δ*dh*PPase to Ap_4_A and greater Δ*H* versus the full-size enzyme (Table 1) suggest that the DHHA2 domain is indeed part of the mechanism that generates tension in CBS-PPase upon Ap_4_A binding. The observation that DHHA2 deletion increased the thermal stability of the remaining domains (Table 4) suggests that the CBS-PPase structure is more relaxed in the absence of the DHHA2 domain than in its presence, which is consistent with the concept of internal inhibition.

The fact that AMP/Mant-AMP binding is not similarly strengthened by the deletion may mean that AMP induces tension in the *dh*PPase structure through its different element, not the DHHA2 domain. This explanation seems likely in view of the different modes of AMP and Ap_4_A binding—the former ligand binds to one subunit, whereas the latter interacts with two subunits [18]. The fact that the AMP effect is not associated with the DHHA2 domain is evidenced by the unchanged *T*_m_ for this domain (peak I) upon AMP binding to the full-size enzyme (Table 4). However, the identity of the structural element that mediates the AMP signal can only be conjectured. Earlier mutagenesis studies of *dh*PPase have identified two interacting elements, helix α1 of CBS2 domain and the loop formed by residues 310–323 in the DHH domain, among the best candidates for signal transmission from the regulatory site to the catalytic DHH domain [37]. Notably, an Ala substitution for the highly conserved Asn312 in this loop reversed the effect of ATP on *dh*PPase from activation to inhibition [37]. Furthermore, replacement of Arg168 in the highly conserved RyR motif of *Moorella thermoacetica* CBS-PPase (corresponding to Arg276 of *dh*PPase) weakened AMP binding and reversed its effect from inhibition to activation [38]. Therefore, both residues may control tension generation in *dh*PPase upon AMP/Mant-AMP binding, and their presence in Δ*dh*PPase may explain the unchanged Mant-AMP binding to it.

Further studies are clearly needed to test these intriguing possibilities and delineate the regulation mechanism of CBS-PPase and other CBS domain-containing enzymes and transporters. These studies will necessarily include the determination and analysis of the 3D structure of full-size CBS-PPase, both empty and with bound regulatory ligands.

## 4. Materials and Methods

### 4.1. Materials

Wild-type *dh*PPase (UniProtKB: B8FP42) and its deletion variant were produced in *E. coli* BL21 cells transformed with the pET-42b vector (Novagen, Merck Group, Darmstadt, Germany) carrying the corresponding genes [19]. The DHHA2 domain (residues 425–544) was deleted in the *dh*PPase gene using overlap extension PCR with Phusion DNA polymerase with the forward/reverse primers TATACATATGTCAAAAAAAATCCATGTCG/TAATCTCGAGTTAAGCTGCCTTGAACATTTG. The isolation procedure included cell disruption by freezing/thawing, ion exchange, and size exclusion chromatography [19]. The purity of the isolated proteins, as estimated by SDS-PAGE [39] with Coomassie staining, was >90%. Protein concentrations in milligrams per milliliter were determined spectrophotometrically, using an *A*^0.1%^_280_ value of 0.477 for *dh*PPase and 0.556 for Δ*dh*PPase, as calculated from the amino acid composition with ProtParam (https://web.expasy.org/protparam/ (accessed on 26 November 2023)). Molar concentrations were calculated in terms of the subunit using subunit molecular masses of 60.5 and 46.6 kDa for *dh*PPase and Δ*dh*PPase, respectively.

P1,P4-Diadenosine 5′-polyphosphate (Ap_4_A, ammonium salt) and AMP (free acid) were obtained from Sigma-Aldrich (St. Louis, MO, USA). 2′/3′-(N-Methyl-anthraniloyl)-adenosine-5´-monophosphate (Mant-AMP, triethylammonium salt) was a Jena Bioscience GmbH (Jena, Germany) product. The concentrations of nucleotide stock solutions were estimated by measuring absorbance at 259 nm (*ε* = 15,900 M^−1^ cm^−1^ for the monoadenosine phosphates and 31,800 M^−1^ cm^−1^ for Ap_4_A).

### 4.2. Enzyme Activity Assay

The initial rates of PP_i_ hydrolysis were measured using a continuous P_i_ assay [40]. The assay medium contained 0.1 M Tes-KOH, pH 7.2, 5.23 mM MgCl_2_, 140 μM PP_i_, (corresponding to 50 µM MgPP_i_ complex). The reaction was initiated by adding 0.3 nM enzyme and continued for 2–3 min at 25 °C. Rate values were obtained from the initial slopes of the P_i_ accumulation curves.

### 4.3. Isothermal Titration Calorimetry (ITC)

Heat production upon nucleotide binding to *dh*PPase and Δ*dh*PPase was measured at 25 °C using a VP-iTC calorimeter (MicroCal Ltd, Piscataway, NJ, USA). Enzyme and adenine nucleotide solutions were prepared on 0.1 M MOPS/KOH (pH 7.2) buffer containing 2 mM MgCl_2_, 0.1 mM CoCl_2_, and 150 mM KCl. Titrations were performed by successive 10-μL injections of 100 μM AMP or 33 μM Ap_4_A solution into 1.4 mL of 8–12 μM protein solutions. The interval between injections was 5 min. The measured heat values were corrected for ligand dilution effects.

### 4.4. Förster Resonance Energy Transfer (FRET) Measurements

The increase in Mant-AMP fluorescence due to FRET from excited protein tryptophanes was recorded in a Cary Eclipse spectrofluorometer (Varian, Inc., Palo Alto, CA, USA) using a 3 × 3 mm optical cuvette. The excitation and emission wavelengths were 295 and 455 nm, respectively, and the slits were set at 5 nm. Portions of 1 or 10 mM Mant-AMP solution were added to 0.3 mL of 5 μM enzyme solution in 0.1 M Mops-KOH buffer (pH 7.2) containing 150 mM KCl, 2 mM MgCl_2_, and 0.1 mM CoCl_2_. The mixture was equilibrated for 1 min after each addition before fluorescence was read. The fluorescence values averaged over a 0.5 min time span were corrected for nucleotide and inner filter effects.

### 4.5. Binding Data Treatment

The ITC data were analyzed using a MicroCal ITC subroutine in Origin 7.0 software (OriginLab, Northampton, MA, USA) using a single binding site model.

The FRET titration curves were analyzed in terms of Scheme 1, which describes the cooperative binding of ligand N to two initially identical sets of binding sites on protein E. *K*_N1_ and *K*_N2_ are the “microscopic” nucleotide-binding constants.

A system of Equations (1)–(5), derived from Scheme 1, was fit to the binding data using SCIENTIST (MicroMath Scientific Software, Salt Lake City, UT, USA). Δ*F* is the fluorescence change, Δ*F*_max_ is its limiting value at an infinite concentration of N, *K*_N1_ and *K*_N2_ are the dissociation constants, and [N] is the free (unbound) ligand concentration. Equations (2) and (3) define the binding constants, and Equations (4) and (5) describe the mass balance for the protein and ligand. This treatment considers a decrease in free ligand concentration because of complex formation. The factor ½ in Equation (1) signifies that all binding sites contribute equally to the fluorescence change upon ligand binding. The total enzyme concentration, [E]_0_, and the concentrations of various enzyme species in Equations (1)–(5) refer to the subunit concentrations; [N]_0_ is the total ligand concentration.

Δ*F* = Δ*F*_max_([EN]/2 + [EN_2_])/[E]_0_ (1)

[E][N]/[EN] = *K*_N1_ (2)

[EN][N]/[EN_2_] = *K*_N2_ (3)

[E] + 2[EN] + [EN_2_] = [E]_0_ (4)

[N] + 2[EN] + 2[EN_2_] = [N]_0_ (5)

Enzyme inhibition data were analyzed in a similar way but using Equation (6) instead of Equation (1) and the respective inhibition constants *K*_i1_ and *K*_i2_ instead of *K*_N1_ and *K*_N2_.

*A* = {*A*_0_[E] + (*A*_0_ + *A*_lim_)[EN]/2 +*A*_lim_[EN_2_]}/[E]_0_ (6)

### 4.6. Protein Thermal Stability Measurements (ThermoFluor)

The instrumental setup consisted of a C1000 thermal cycler with a CFX96 real-time PCR detection system (Bio-Rad, Hercules, CA, USA). The assay mixtures (50 μL volume), placed in transparent low-profile 96-well multiplate PCR plates (SSIbio, Lodi, CA, USA), contained 5–10 μM protein, 0.02% SYPRO Orange dye (Invitrogen-Thermo Fisher, Waltham, MS, USA), 0.1 M Mops-KOH buffer (pH 7.2, measured at 25 °C), 2 mM MgCl_2_, 0.1 mM CoCl_2_, and 150 mM KCl. The indicated adenine nucleotides were added at the following concentrations: Ap_4_A, 20 µM; AMP, 200 µM. These concentrations exceeded 100–1000-fold the dissociation constants for the nucleotide complexes of *dh*PPase [6,15]. The plates were covered with an UltraFlux Standard PCR sealing film (SSIbio) and heated from 30 to 95 °C with stepwise increments of 0.5 °C and a 40 s hold step for every point. Fluorescence was excited at 515–535 nm and monitored at 560–580 nm (second channel, “VIC” setting), and melting temperatures, *T*_m_, were derived from the resulting curves using the BioRad CFX Manager 3.1 software.

### 4.7. Size-Exclusion Chromatography

Protein samples (0.5 mL, 5–10 mg protein) in elution buffer (0.1 M Mops-KOH, pH 7.2, containing 150 mM KCl, 2 mM MgCl_2_, and 0.1 mM CoCl_2_), were applied onto a calibrated Superdex 200 Increase 10/300 GL column (GE HealthCare, Chicago, IL, USA) equilibrated with the same buffer. The elution was performed on a GE HealthCare AKTA Purifier 100 FPLC System at a flow rate of 0.4 mL/min, with absorbance monitoring at 280 nm. Fractions (0.5 mL) were collected.

### 4.8. Sedimentation Velocity Analysis

Analytical ultracentrifugation was performed at 25 °C in a Spinco E instrument (Beckman Instruments, Fullerton, CA, USA) equipped with a computerized data collection unit, with scanning at 280 nm. Samples containing 10 μM protein in the isolation buffer were preincubated for 6 h at 25 °C. The sedimentation velocity was measured at 60,000 rpm, and the sedimentation coefficients (*s*_20,w_) and molecular masses were estimated using the program SedFit [23].

### 4.9. Structure Modeling and Refinement

The three-dimensional structure of the dhPPase deletion variant was predicted from its amino acid sequence using AlphaFold2 (version 2.3.0) [25] with default parameter settings. All calculations were performed using Nvidia (Santa Clara, CA, USA) RTX A5000 graphical card. Twenty five models generated (five models per prediction mod) were ranked according to their iptm + ptm score, and the best model (score = 0.807) was selected.

### 4.10. Single-Particle Electron Microscopy

Samples for negatively stained transmission electron microscopy (TEM) were prepared from 3 μL of 2.5 mg/mL (52 µM) Δ*dh*PPase solution in 0.1 M MOPS-KOH buffer, pH 7.2, containing 150 mM KCl, 2 mM MgCl_2_, and 0.1 mM CoCl_2_. The protein samples were placed on a glow-discharged carbon film (Ted Pella, Redding, CA, USA), blotted after 60 s, and stained with two droplets of 1% uranyl acetate solution for 10 and 20 s. The data for single particle analysis were collected using a JEOL JEM-2100 200 keV transmission electron microscope equipped with a Direct Electron DE20 camera. Approximately 70,000 particle projections were collected and subjected to 2D classification in cryoSPARC [41].

Samples for cryo-EM were prepared from 3 μL of 2.5 mg/mL Δ*dh*PPase solution in 0.1 M MOPS-KOH buffer, pH 7.2, containing 150 mM KCl, 2 mM MgCl_2_, and 0.1 mM CoCl_2_. The protein samples were placed on a glow-discharged 300 mesh lacey carbon film on Copper TEM Grids (Ted Pella, Redding, CA, USA), blotted, and vitrified using an EM GP2 Automatic Plunge Freezer from Leica Microsystems CMS GmbH (Wetzlar, Germany) according to the standard procedure [42]. The vitrification parameters were as follows: 4 °C, 95% humidity in the specimen chamber, 30 s pre-blot time, 12 s blot time with Whatman #1 filter paper, and immediate vitrification in liquid ethane at -180 °C. A Gatan (Pleasanton, CA, USA) Elsa Cryotransfer Holder was used to acquire the cryoEM dataset using a JEOL (Akishima, Tokyo, Japan) JEM-2100 200 keV electron microscope. SerialEM [43] was used to acquire 800 stacks with a 2 µm defocus and 60e/Å^2^ total electron dose.

Further data processing was performed in cryoSPARC [41]. Initially, 737 image stacks were subjected to motion correction and patch contrast transfer function (CTF) estimation. The images were manually inspected to exclude views with aggregates and crystalline ice. Blob-based auto picking was performed, followed by 2D classification and a subsequent run of template-based autopicking. A total of 52,000 particle projections were picked, and 15,000 were left after the 2D classification. An initial reference was generated with a cryoSPARC, version 4.4.0+231114 (https://cryosparc.com/ (accessed on 26 November 2023)) ab-initio approach, and final reconstruction was performed using non-uniform refinement. Reprocessing and blob autopicking were performed using the standard procedure.

The AlphaFold2 model was flexibly fitted into the density map using coot of the CCP4 Software suit, version 8.0.016 (https://www.ccp4.ac.uk/ (accessed on 26 November 2023)). A set of interatomic distance restraints with 5.0 Å radius was derived from the initial model. The model underwent real-space refinement for all chains with the Geman-McClure alpha parameter set to 0.01.

## Figures and Tables

**Table 1:** Thermodynamic parameters for the complexes of *dh*PPase with Ap_4_A and AMP, as obtained by isothermal calorimetry.

Enzyme Variant	Nucleotide	Δ*H*, kcal/mol	*n*	*K*_N_, µM
Δ*dh*PPase	Ap_4_A	−17.0 ± 0.3	0.45 ± 0.01	<<0.01
*dh*PPase	Ap_4_A	−10.8 ± 0.2 (−10.4 ± 0.3) ^a^	0.42 ± 0.01 (0.41 ± 0.01) ^a^	0.01–0.1
*dh*PPase ^a^	AMP	−5.6 ± 0.5	0.79 ± 0.05	0.8 ± 0.3

^a^ From Ref. [6].

**Table 2:** Comparison of Mant-AMP and AMP as inhibitors of *dh*PPase.

Nucleotide	Residual Activity, %	*h*	*K*_i1_, µM	*K*_i2_, µM
Mant-AMP	<1	1.75 ± 0.04	41 ± 7	0.8 ± 0.1
AMP ^a^	3.7 ± 0.1	1.68 ± 0.04	9.6 ± 1.4	0.21 ± 0.08

^a^ From Ref. [15].

**Table 3:** The parameter values for Mant-AMP binding derived from the data in Figure 3B.

Enzyme Variant	Δ*F*_max_	*K*_N1_, µM	*K*_N2_, µM
*dh*PPase	10.3 ± 0.4	22 ± 2	6.5 ± 1
Δ*dh*PPase	8.8 ± 0.4	21 ± 6	6 ± 2

**Table 4:** The melting temperatures of full-size *dh*PPase and its deletion variants and the effects of adenosine phosphates. The values are precise within 0.5 °C.

Nucleotide	*T*_m_, °C			
	*dh*PPase	Δ*dh*PPase	CDC	ΔΔ*dh*PPase
None	57.0, 64.0, 77.5	68.3, 83.5	58.0	56.5, 77.5
AMP	57.1, 67.4, 80.8	68.6, 85.5	66.5	
Ap_4_A	52.9, 68.8, 79.7	71.0, 86.5	61.5	

**Table 5:** The molecular masses of *dh*PPase and its deletion variant.

Enzyme Variant	Molecular Mass, kDa		
	Sedimentation ^a^	SEC	Theory ^b^
Δ*dh*PPase	171–192	~176	186
*dh*PPase ^c^	203–234	~214	241

^a^ The ranges of the masses shown were estimated assuming frictional ratios of 1.25–1.35, typical for globular proteins [23]. ^b^ Calculated from the amino acid sequence. ^c^ From Ref. [19].

## Data Availability

The cryo-EM map of ΔdhPPase is available as EMD-19687 in the EMDB database. Other data are available on request from the corresponding author.

## Supplementary Materials

The following supporting information can be downloaded at: https://www.mdpi.com/article/10.3390/ijms242417160/s1.
