# Supplementary material for: The Structure and Nucleotide-Binding Characteristics of Regulated Cystathionine β-Synthase Domain-Containing Pyrophosphatase without One Catalytic Domain
Source: Int J Mol Sci. 2023 Dec 5;24(24):17160. doi: 10.3390/ijms242417160 (PMC10742508; doi:10.3390/ijms242417160)
Supplement: Supplementary file 1 [file ijms-24-17160-s001.zip › ijms-2720705-supplementary.pdf]

Supplementary Material for:

"The structure and nucleotide-binding characteristics of regulated CBS domain-containing pyrophosphatase without one catalytic domain"

Ilya M. Zamakhov, Viktor A. Anashkin, Andrey V. Moiseenko, Victor N. Orlov, Natalia N. Vorobyeva, Olga S. Sokolova, Alexander A. Baykov

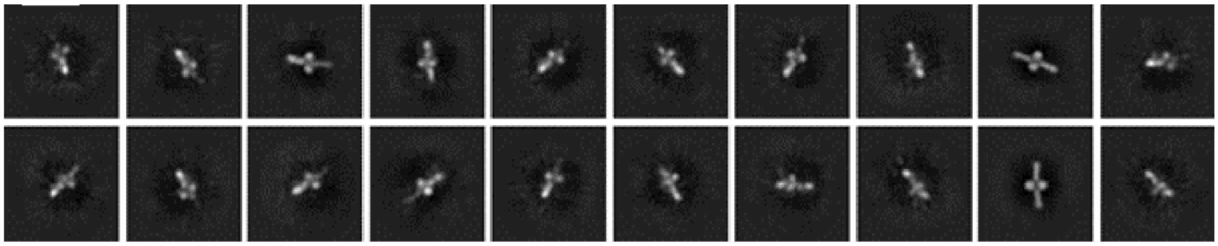

Figure S1. Representative 2D class average images of negatively stained Δ*dh*PPase.

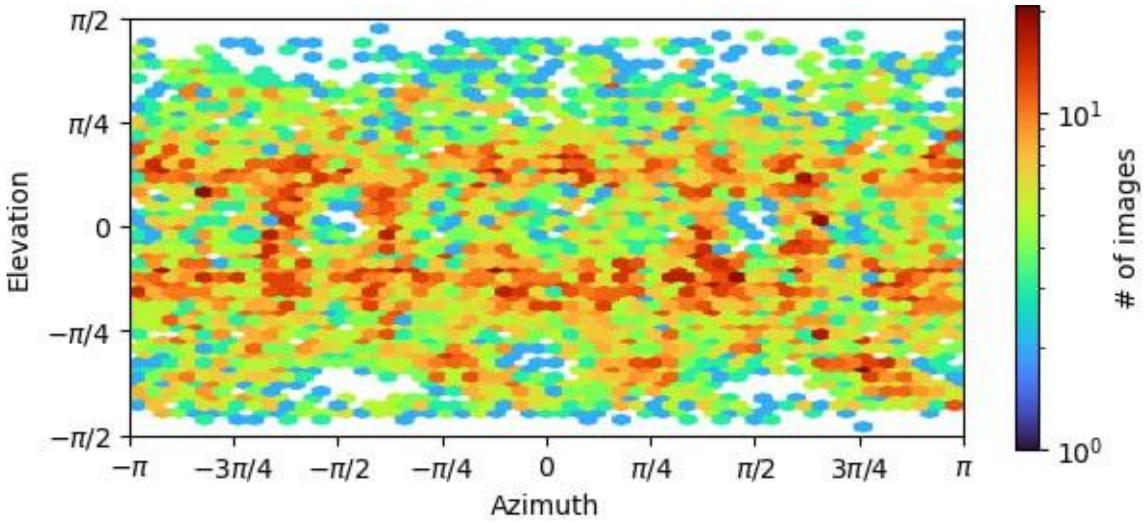

Figure S2. The angular distribution of particle projections obtained by cryo-EM.

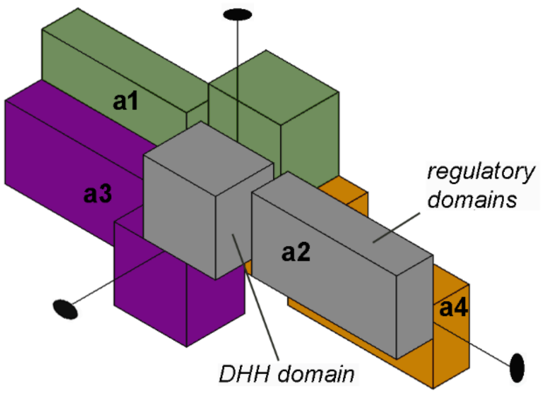

Figure S3. A schematic view of tetrameric Δ*dh*PPase organization in the modeled structure. Subunits containing DHH domain and regulatory domains (CBS1, CBS2, and DRTGG), are depicted in different colors. Three symmetry axes corresponding to D2 symmetry are shown.
